# Clinical validation of the nursing diagnosis “Risk for delayed child development”^*^

**DOI:** 10.1590/1980-220X-REEUSP-2022-0229en

**Published:** 2022-12-16

**Authors:** Nádia Proença de Melo, Juliana Martins de Souza, Samara Macedo Cordeiro, Maria de La Ó Ramallo Veríssimo

**Affiliations:** 1 Universidade Nove de Julho, São Paulo, SP, Brazil.; 2 Universidade Federal de Catalão, Catalão, GO, Brazil.; 3 Faculdade de Ciências Médicas da Santa Casa de São Paulo, São Paulo, SP, Brazil.; 4 Universidade de São Paulo, Escola de Enfermagem, São Paulo, SP, Brazil.

**Keywords:** Child Developmen, Validation Stud, Pediatric nursin, Nursing Diagnosi, Terminology, Desarrollo Infantil, Estudio de Validación, Enfermería Pediátrica, Diagnóstico de Enfermería, Terminología, Desenvolvimento Infantil, Estudo de Validação, Enfermagem Pediátrica, Diagnóstico de Enfermagem, Terminologia

## Abstract

**Objective:**

To validate clinically the risk factors of the nursing diagnosis “Risk for delayed child development”.

**Method:**

Cross-sectional quantitative study carried out in a specialty outpatient clinic and in family health units with 124 children. The data was collected through interviews with the children’s guardians to investigate the risk factors for delay in child development.

**Results:**

The tested risk factors affected 108 of the evaluated children (87.1%). In the accuracy tests, most specificity values were above 80% and sensitivity values were lower than 30%. Most risk factors had odds ratio >1, three of which were noteworthy: genetic disorder (OR = 38, p < 0.05) and congenital disorder (OR = 4.4, p < 0.05), among child-related aspects, and impaired cognitive development in parents (OR = 27, p < 0.05), among caregiver-related aspects.

**Conclusion:**

The study contributed to a refined diagnostic accuracy, identifying potential associated factors of the evaluated diagnosis.

## RESUMO

**Objetivo:**

Realizar a validação clínica dos fatores de risco do diagnóstico de Enfermagem “Risco de atraso no desenvolvimento infantil”.

**Método:**

Estudo transversal, de abordagem quantitativa, realizado em um ambulatório de especialidades e em unidades de saúde da família, com 124 crianças. A coleta de dados ocorreu por meio de entrevistas com os responsáveis pelas crianças e investigou os fatores de risco para atraso no desenvolvimento infantil.

**Resultados:**

Os fatores de risco sob teste estiveram presentes em 108 crianças avaliadas (87,1%). Nos testes de acurácia, a maioria dos valores de especificidade foi acima de 80%, e os de sensibilidade, inferiores a 30%. A maioria dos fatores de risco teve odds ratio >1, com destaque para três: distúrbio genético (OR = 38, p < 0,05) e distúrbio congênito (OR = 4,4, p < 0,05), entre os aspectos relativos à criança, e o desenvolvimento cognitivo dos pais prejudicado (OR = 27, p < 0,05), entre os aspectos dos cuidadores.

**Conclusão:**

O estudo contribuiu para o refinamento da acurácia diagnóstica, identificando fatores potencialmente associados ao diagnóstico avaliado.

## RESUMEN

**Objetivo:**

Realizar la validación clínica de los factores de riesgo del diagnóstico de enfermería “Riesgo de retraso en el desarrollo infantil”.

**Método:**

Estudio transversal, cuantitativo, realizado en un ambulatorio de especialidades y en unidades de salud de la familia, con 124 niños. Los datos se recogieron a través de entrevistas con los tutores de los niños y se investigaron los factores de riesgo para el retraso en el desarrollo infantil.

**Resultados:**

Los factores de riesgo sometidos a prueba estuvieron presentes en 108 de los niños evaluados (87,1%). En las pruebas de precisión, la mayoría de los valores de especificidad fueron superiores al 80% y los valores de sensibilidad fueron inferiores al 30%. La mayoría de los factores de riesgo tenían *odds ratio* >1, con énfasis en tres: el trastorno genético (OR = 38, p < 0,05) y el trastorno congénito (OR = 4,4, p < 0,05), entre los aspectos relacionados con el niño, y el desarrollo cognitivo deficiente de los padres (OR = 27, p < 0,05), entre los aspectos de los cuidadores.

**Conclusión:**

El estudio contribuyó para el refinamiento de la precisión diagnóstica, identificando factores potencialmente asociados con el diagnóstico evaluado.

## INTRODUCTION

An effective development in the first years of life enables the formation of safer individuals, capable of facing adverse life situations, limiting economic and social gaps^(1)^; therefore, favoring conditions for an adequate development may be what matters the most in this phase of life^(2)^. Nurses are thus responsible for diagnoses related to the needs of the development process, in addition to those targeted at potential health changes.

The safe application of diagnoses in nursing practice depends on their validation; being valid means that a diagnosis is based on evidence and theories that support its clinical use^(3)^. In this sense, this study presents the clinical validation of a diagnosis related to child development (CD), given its importance in nursing practice to promote children’s health.

NANDA-I has been demonstrating the need to review nursing diagnoses (ND) to achieve levels of evidence with more robust validation methods based on epidemiological approaches and establishing the accuracy of clinical indicators and causal relationships, among others^(3)^. There is an increasing number of studies attempting to use new methods of clinical validation of diagnoses, such as accuracy studies, since they correctly identify whether a diagnosis applies to an individual^(4)^.

Diagnoses are essential components of the nursing process due to enabling the classification of the evaluated situation and guiding the definition of interventions^(3)^. Validated diagnoses offer nurses a set of uniform, safe, and reliable elements for this classification^(4)^ and contribute to quality childcare^(5)^.

A study demonstrated that the main classifications used in Brazilian nursing, CIPE and NANDA-I, did not address the phenomenon of CD in its complexity^(6)^. The definitions of NANDA-I diagnoses had limitations, hindering their application, and addressed different concepts, growth and development, in a single diagnosis. Such diagnoses were not validated and were excluded in the 2015 edition^(7)^ of the taxonomy.

To fill this gap, a study based on the concept analysis of the phenomenon of CD^(8)^, based on the Bioecology of Human Development^(9)^, proposed new diagnoses related to early childhood CD^(10)^. These diagnoses were evaluated by judges and experts on this subject with high levels of agreement^(8,11)^.

Aiming at a clinical validation of the nursing diagnosis “Risk for delayed child development”^(10)^ proposed for the NANDA-I taxonomy, this study had the general objective of clinically validating the risk factors of the nursing diagnosis “Risk for delayed child development”^(10)^ and the specific objectives of testing the measures of accuracy and the association of risk factors with the proposed diagnosis.

## METHODS

### Type of Study

Descriptive, cross-sectional, quantitative, non-experimental study, excerpted from a master’s dissertation^(12)^. The presentation of the study follows the Strengthening the Reporting of Observational Studies in Epidemiology (Strobe) instrument.

### Time and Local of Study

The data was collected from June to October 2017 in two scenarios: a specialty outpatient clinic of a public children’s hospital located in the municipality of São Paulo, state of São Paulo, and in primary care in the municipality of Catalão, state of Goiás. This choice aimed at covering a greater diversity of individual and social conditions of children and understanding how the components of the diagnosis resembled and differed from frequent childcare-related situations in that context.

### Population and Sample

The study included children aged 0 to 3 years whose parents or parents agreed to participate. The excluded participants were children in unstable health conditions at the time of collection and caregivers or parents who did not have information about pregnancy, delivery, and childcare.

The sample calculation was performed by a statistician based on a pilot test with 35 children in the specialty outpatient clinic, following the study protocol. Considering the classification of lower prevalence according to the child’s health booklet^(13)^, which was “probable delayed child development” the sample was defined as 112 children, 56 for each center. As there were no changes in the instruments and collection procedures, the pilot data was included in the final sample, according to the research project.

### Study Protocol

Clinical validation studies enable testing, in practice, the attributes proposed by the taxonomy review during concept analysis and content validation by specialists, steps which were previously followed, as mentioned in the introduction^(4)^. Such analyses should measure the accuracy, or sensibility and specificity, of clinical constructs^(4,14)^.

The existence of a gold standard of comparison enables measuring how representative a diagnosis is, and its absence is considered a limitation^(14)^. In this study, the CD surveillance instrument in the children’s health booklet^(13)^, recommended by the Brazilian Ministry of Health for childcare consultations, was adopted as a reference.

The data was collected through semi-structured interviews with parents or caregivers in both study scenarios or in home visits. They were guided by a data collection plan elaborated through the theoretical framework^(8,11)^, addressing the variables listed as risk factors. The 56 questions investigated the children’s health, pregnancy, and birth records; daily care; parental occupation; household; and the analysis of the evaluation of child development.

The collected data aimed to identify the 22 risk factors of the diagnosis under study, organized into: child risk factors; aspects related to pregnancy; aspects related to daily care. The child’s risk factors were: (acute and chronic) diseases; genetic disorders; congenital disorders; sensory disorders; inadequate growth; prematurity and/or low birth weight. The following aspects related to pregnancy were addressed: use of medications during pregnancy; tobacco use during pregnancy; use of alcohol and drugs during pregnancy; exposure to environmental pollutants (e.g., nitrogen dioxide, benzene, lead, manganese, pesticides, heavy metals); altered maternal mental health during pregnancy; maternal disease; and insufficient prenatal follow-up. Aspects related to daily care were exposure to domestic violence; impaired cognitive development of parents; institutionalization; lack of child stimulation; unfavorable social conditions; and unfavorable economic conditions.

To evaluate the variables “Unfavorable social conditions” and “Unfavorable economic conditions”, the questions of the Social Reproduction Index^(15)^ form were used. This index divides the population into four social groups, not restricted to the purchasing power of families, but considering their insertion in the means of production and social reproduction. The conditions of the families classified between groups 1 and 2 were considered “favorable” and those in groups 3 and 4, “unfavorable”. The choice of this index considered the complexity of the child development phenomenon and the importance of the care environment, which is not defined only by purchasing power.

A data collection manual was elaborated, including the description of the interview’s steps, contents to be investigated, and orientation for the evaluation of development. The operational definitions of the manual standardized the collection in both sites and ensured the reliability and validity of the study. The interviewers, one for each collection site, were trained together, with guidance from two experienced researchers.

After the application of the script, CD was evaluated according to the child’s health booklet^(13)^. At the end of the interview, the parents received feedback on CD assessment and the developmental surveillance instrument was filled out in the child’s health booklet.

### Data Analysis

Data analysis began during the interview, with the classification of each child’s CD, as defined in the health booklet^(13)^, i.e., adequate development, adequate development with risk factors, developmental concern or probable developmental delay, as feedback to parents. Based on these classifications, the children were divided into two groups: children who achieved all the milestones of their age group, classified with “adequate development”^(13)^ or “adequate development with risk factors”^(13)^; and children who did not present the milestones of their age group, classified with “developmental concern”^(13)^ or “probable delayed development”^(13)^.

The data were then transferred to the SPSS program (version 22) to verify the prevalence of risk factors of the diagnosis under test and to apply the measures of accuracy and measures of association to two groups: children with absent developmental milestones and with present developmental milestones.

To evaluate the measures of accuracy of risk factors, the measures of Sensitivity (SE), Specificity (SP), Positive (PPV) and Negative Predictive Value (NPV) were calculated. The values of specificity and sensitivity were classified according to the standards used in studies that verify the accuracy of diagnoses^(16,17)^. Pearson’s Chi-Square and Fischer’s Test with significance of p < 0.05 were applied to evaluate the measures of association.

### Ethical Aspects

This study, conducted with human beings, complies with Resolution No. 466/12 and included a consent form (Opinion 2,070,709, approved in 2017).

## RESULTS

The interviews lasted 30 to 60 minutes. The study included 124 children, 59 from the specialty outpatient clinic and 65 from primary healthcare units; 50 (40.3%) children were aged 0–12 months, 39 (31.4%) were 12–24 months old, and 35 (28.2%) were 24–36 months old; 72 (58.0%) were female.

The most noteworthy risk factors included unfavorable social and economic conditions, chronic acute diseases, congenital disorders, inadequate growth, and prematurity or low birth weight (Table 1).

**Table 1.** Distribution of the risk factors proposed for the diagnosis “Risk for Delayed Child Development” in the study sample – São Paulo, 2018 (N = 124).

Risk factors	Present N (%)	Absent N (%)
Chronic diseases	15 (12.1)	109 (87.9)
Acute diseases	25 (20.2)	99 (79.8)
Genetic disorders	8 (6.5)	116 (93.5)
Congenital disorders	29 (23.4)	95 (76.6)
Sensory disorders	2 (1.6)	122 (98.4)
Inadequate growth	28 (22.5)	96 (77.5)
Prematurity and/or low birth weight	24 (19.4)	100 (80.6)
Use of medications during pregnancy	1 (0.8)	123 (99.2)
Tobacco use during pregnancy	12 (9.7)	112 (90.3)
Use of alcohol and drugs during pregnancy	11 (8.9)	113 (91.1)
Exposure to environmental pollutants	1 (0.8)	123 (99.2)
Altered maternal mental health during pregnancy	9 (7.3)	115 (92.7)
Maternal disease	6 (4.8)	118 (95.2)
Insufficient prenatal follow-up	6 (4.8)	118 (95.2)
Bond with the impaired caregiver	1 (0.8)	123 (99.2)
Exposure to domestic violence	9 (7.3)	115 (92.7)
Impaired cognitive development of parents	3 (2.4)	121 (97.6)
Lack of support from the health professional	11 (8.9)	113 (91.1)
Institutionalization	3 (2.4)	121 (97.6)
Lack of child stimulation	2 (1.6)	122 (98.4)
Unfavorable social conditions	41 (33.1)	83 (66.9)
Unfavorable economic conditions	34 (27.4)	90 (72.6)

Based on the health booklet^(13)^, the children were classified with: adequate development – 44 (35.4%), adequate development with risk factors – 55 (44.3%), developmental concern – 12 (9.6%) and probable delayed development – 13 (10.4%). Thus, the group with developmental milestones comprised 99 children (79.8%) and the group with missing developmental milestones was formed by 25 children (20.2%). The distribution of risk factors of the proposed diagnosis between these two groups is presented in Table 2.

**Table 2.** Distribution of risk factors of the diagnosis “Risk for delayed child development” (proposed by NANDA-I), according to the groups with absent developmental milestones and with present developmental milestones – São Paulo, 2018 (N = 124).

Risk factors	Children with developmental milestones N (%)	Children with missing developmental milestones N (%)
Chronic diseases	11 (73.3)	4 (26.7)
Acute disease	21 (84.0)	4 (16.0)
Genetic disorders	1 (12.5)	7 (87.5)
Congenital disorders	17 (58.6)	12 (41.3)
Sensory disorders	1 (50.0)	1 (50.0)
Inadequate growth	24 (85.5)	4 (14.2)
Prematurity and/or low birth weight	16 (66.6)	8 (33.3)
Use of medications during pregnancy	1 (100)	0 (0.0)
Tobacco use during pregnancy	8 (66.6)	4 (33.3)
Use of alcohol and drugs during pregnancy	9 (81.8)	2 (18.2)
Exposure to environmental pollutants	1 (100)	0 (0.0)
Altered maternal mental health during pregnancy	5 (55.5)	4 (44.4)
Maternal disease	6 (100)	0 (0.0)
Insufficient prenatal follow-up	4 (66.6)	2 (33.3)
Exposure to domestic violence	9 (100)	0 (0.0)
Impaired cognitive development of parents	0 (0.0)	3 (100)
Lack of support from the health professional	10 (90.9)	1 (11.1)
Institutionalization	2 (66.6)	1 (33.3)
Lack of child stimulation	1 (50.0)	1 (50.0)
Unfavorable social conditions	32 (78.0)	9 (21.9)
Unfavorable economic conditions	31 (91.2)	3 (8.8)

All risk factors of the diagnosis under test were present in both groups of children. Some maternal and environmental conditions did not appear in the group of children with missing milestones, and the variable “impaired cognitive development of parents” surfaced only in this group.

Table 3 presents the test of the attributes of risk factors in clinical practice, that is, the measures of accuracy of risk factors of the nursing diagnosis under evaluation.

**Table 3.** Distribution of accuracy tests for the variables of diagnostic “Risk for delayed CD” – São Paulo, 2018 (N = 124).

Risk factors	Specificity (%)	Sensitivity (%)	Positive predictive value (%)	Negative predictive value (%)
Chronic diseases (n = 15)	88.9	16.0	26.7	80.7
Acute disease (n = 25)	78.8	16.0	16.0	78.8
Genetic disorders (n = 8)	99.0	28.0	87.5	84.5
Congenital disorders (n = 29)	82.8	48.0	41.4	86.3
Sensory disorders (n = 2)	99.0	4.0	50.0	80.3
Inadequate growth (n = 28)	75.8	16.0	14.3	78.1
Prematurity and/or low birth weight (n = 24)	83.8	32.0	33.3	83.0
Use of medications during pregnancy (n = 1)	99.0	0.0	0.0	79.7
Tobacco use during pregnancy (n = 12)	91.9	16.0	33.3	81.3
Use of alcohol and drugs during pregnancy (n = 11)	90.9	8.0	18.2	79.6
Exposure to environmental pollutants (n = 1)	99.0	0.0	0.0	79.7
Altered maternal mental health during pregnancy (n = 9)	94.9	16.0	44.4	81.7
Maternal disease (n = 11)	93.9	0.0	0.0	78.8
Insufficient prenatal follow-up (n = 6)	96.0	8.0	33.3	80.5
Exposure to domestic violence (n = 9)	90.9	0.0	0.0	78.3
Impaired cognitive development of parents (n = 3)	100.0	12.0	100.0	81.8
Lack of support from the health professional (n = 11)	89.8	4.0	10.1	90.9
Institutionalization (n = 3)	98.0	4.0	33.3	80.2
Lack of child stimulation (n = 2)	99.0	4.0	50.0	80.3
Unfavorable social conditions (n = 41)	67.7	36.0	22.0	80.7
Unfavorable economic conditions (n = 34)	68.7	12.0	8.8	75.6

Specificity was mostly above 80%. This indicates that the absence of these factors is high in children with all developmental milestones for their age. Slightly lower specificity was found in four factors: “acute diseases” (78.8%), “inadequate growth” (75.8%), “unfavorable social conditions” (67.7%), and “unfavorable economic conditions” (68.7%). Negative predictive values (NPV) were mostly above 80%, followed by factors above 75%. This data indicates that children who did not meet all CD milestones, according to the booklet, had a higher presence of these risk factors.

Sensitivity values were lower than 30.0% for almost all risk factors and “congenital disorders” was the factor with the highest sensitivity (48.0%). The positive predictive values (PPV) were mostly lower than or equal to 50.0%, except for the risk factor “genetic disorders” (87.5%) and “cognitive development of parents” (100.0%). This means that most of the analyzed risk factors were not associated with the absence of developmental milestones. Thus, children presenting risk factors for delayed CD according to the proposed nursing diagnosis will not necessarily be classified with probable delay or concern based on the child’s booklet^(13)^.

Regarding the measures of association of risk factors of the proposed diagnosis without CD milestones, it was identified that most had odds ratio > 1, emphasizing “genetic disorder” (OR = 38, p < 0.05) and “congenital disorder” (OR = 4.4, p < 0.05) among child-related aspects, and “impaired cognitive development of parents” (OR = 27, p < 0.05), among the caregiver-related aspects. This means that children with these risk factors are more likely not to meet all CD milestones for their age group.

## DISCUSSION

This study performed the clinical validation of the risk factors of the nursing diagnosis “Risk for delayed child development” proposed by NANDA-I through the evaluation of children from 0 to 3 years of age in the primary health network and in a secondary care service.

The children’s health booklet^(13)^ was the instrument for evaluating the reference CD for comparison of the diagnosis under analysis. This is an important and easily accessible instrument to identify development issues and one of its advantages is using risk factors to classify children^(18)^, which was also considered when it was selected for this study.

Although the risk factors included in the classifications of the health booklet^(13)^ cover aspects of the child and the family, these do not include aspects of care and stimulation, nor social and economic conditions, which were pointed out in the literature review as important risk factors for CD^(10,11)^. The wider scope of risk factors of the proposed diagnosis for NANDA-I considers the wide variability of factors that influence CD^(19)^ and favors raising awareness of professionals to aspects that are not always evaluated during the childcare consultation, such as: the care and interactions of caregivers with children, affective bonds, organization and establishment of limits, in addition to the environment in which children grow and develop^(8,20)^. Given that such aspects can produce negative impacts for CD, the inclusion of risk factors in a scoring system for CD evaluation is an innovation that enables the early identification of children with delayed CD^(21,22,23,24)^.

However, there is a need to improve the definition of parameters, the choice of indicators, and the awareness of professionals regarding their application^(23)^. Such concern with the definition of parameters and the choice of risk indicators of the proposed diagnosis guided this validation study.

Regarding the measures of accuracy, specificity values were observed to be higher than those of sensitivity. These results are similar to those found in other studies that used CD assessment instruments^(23,24)^. Although it is desirable that the evaluation instruments, in general, have high values of sensitivity and specificity, in the case of CD, these values are not reached, possibly because aspects of various dimensions are evaluated, which, in isolation, may not damage development. This requires continued and broad child monitoring.

Thus, the use of CD assessment instruments and the classification of the diagnosis of children under 5 years of age is unreliable when performed only once, because children in this age group experience widely varied changes, and frequent evaluations are required during their growth^(19,23)^, since the tests are not predictive and only produce results that classify the situation of a particular moment.

Studies on the use of screening tests for the evaluation of CD have been discussed by some authors and reinforce the use of tools involving expanded aspects of CD^(16,18)^. In this sense, the proposal for child evaluation should focus more than the acquisition of skills, including other aspects related to development, such as the risk factors proposed in this study, in order to make development monitoring integral.

There were three significant statistical associations of risk factors with the absence of CD milestones: “Genetic disorders”, “Congenital disorders”, and “Impaired cognitive development of parents”, similar to research results that emphasize these factors as associated with delayed CD^(19,24,25,26)^. Similarly, genetic and congenital disorders are pointed out as factors that have important neurological consequences for children^(26,27)^.

The wide scope of the CD dimensions corroborates the relevance of expanded interventions to promote development in early childhood. This has been emphasized by international organizations, such as the United Nations Children’s Fund (UNICEF) and the World Health Organization (WHO), and Brazilian laws, such as the Legal Framework for Early Childhood (*Marco Legal para primeira infância*), which advocate actions and involvement from various sectors in order to promote favorable conditions to integral child development in early childhood^(28,29,30)^.

Based on the above, the application of the nursing diagnosis “Risk for delayed child development” in the practice of nurses contributes to the early identification of risk factors for delayed CD and favors effective interventions.

### Study Limitations

Sample size, as well as the single-moment cross-sectional evaluation, may have limited the association of some risk factors with the absence of developmental milestones. In future studies, it is recommended the continued effort to refine the nursing diagnosis “Risk for delayed child development” in order to obtain more clarity regarding the data acquisition and better responses in accuracy tests.

### Implications for Nursing

The clinical validation of a diagnosis ensures a basis and the refinement of the practice of nurses in CD surveillance by encouraging them to attend to risk factors. The use of diagnoses tested in care practice provides the professional with a precise and safe classification of this diagnosis. Nursing diagnoses of the risk of delayed CD covering broad aspects, such as those studied in this research, contribute to an expanded evaluation and performance of nursing regarding child development.

## CONCLUSIONS

This study tested in clinical practice attributes considered as risk factors for the nursing diagnosis “Risk for delayed CD” of the NANDA-I taxonomy. The presence of all risk factors proposed for the diagnosis under study and the application of statistical tests contributed to a refined diagnostic accuracy, identifying three factors associated with the absence of developmental milestones: genetic disorder, congenital disorder, and impaired cognitive development of parents.
